# 5′-hydroxy Auraptene stimulates osteoblast differentiation of bone marrow-derived mesenchymal stem cells via a BMP-dependent mechanism

**DOI:** 10.1186/s12929-019-0544-7

**Published:** 2019-07-05

**Authors:** Basem M. Abdallah, Enas M. Ali

**Affiliations:** 1 Biological Sciences Department, College of Science, King Faisal University, Hofuf-31982, Al-Ahsa, Saudi Arabia; 2 Endocrine Research (KMEB), Department of Endocrinology, Odense University Hospital and University of Southern Denmark, Odense, Denmark; 3 Department of Botany and Microbiology, Faculty of Science, Cairo University, Cairo, Egypt

**Keywords:** Mesenchymal stem cells, BMSCs, Osteoblast, Adipocyte, 5-hydroxy aurapten, Osteoblast differentiation

## Abstract

**Background:**

Identifying bone anabolic agents is a superior strategy for the treatment of osteoporosis. Naturally, derived coumarin derivatives have shown osteoanabolic effect in vitro and in vivo. In this study, we investigated the effect of 5′-Hydroxy Auraptene (5′-HA), a coumarin derivative that newly isolated from *Lotus lalambensis* Schweinf on the differentiation of the mouse bone marrow-derived mesenchymal (skeletal) stem cells (mBMSCs) into osteoblast and adipocyte.

**Methods:**

The effect of 5′-HA on mBMSCs cell proliferation and osteoblast differentiation was assessed by measuring cell viability, quantitative alkaline phosphatase (ALP) activity assay, Alizarin red staining for matrix mineralization and osteogenic gene array expression. Adipogenesis was measured by Oil Red O staining and quantitative real time PCR (qPCR) analysis of adipogenic markers. Regulation of BMPs signaling pathways by 5′-HA was measured by Western blot analysis and qPCR.

**Results:**

5′-HA showed to stimulate the differentiation of mBMSCs into osteogenic cell lineage in a dose-dependent manner, without affecting their differentiation into adipocytic cell lineage. Treatment of mBMSCs with 5′-HA showed to promote significantly the BMP2-induced osteogenesis in mBMSCs via activating Smad1/5/8 phosphorylation and increasing Smad4 expression. Blocking of BMP signaling using BMPR1 selective inhibitor LDN-193189 significantly inhibited the stimulatory effect of 5′-HA on osteogenesis.

**Conclusions:**

Our data identified 5′-HA, as a novel coumarin derivative that function to stimulate the differentiation of mBMSCs into osteoblasts in BMP-signaling dependent mechanism.

**Electronic supplementary material:**

The online version of this article (10.1186/s12929-019-0544-7) contains supplementary material, which is available to authorized users.

## Introduction

Osteoporosis is an endocrine-metabolic bone disease, characterized by reduced bone mass, strength, and microarchitecture, which increases the propensity of fragility fractures [1]. The current drug therapy for osteoporosis is mainly based on anti-resorptive drugs, that inhibit osteoclastic bone resorption e.g. bisphosphonates, estrogen agonists/antagonists, and recently, Denosumab (a key osteoclast cytokine, a monoclonal antibody for receptor activator of NF-κB ligand, RANKL) [2]. On the other hand, less anabolic drugs are available for osteoporosis, except for parathyroid hormone that act to enhance bone formation with an adverse effect of increase bone resorption in long term treatment [3, 4]. Thus, there is a need for developing new anabolic bone drugs due to their fast action in the treatment of osteoporosis, as well as their potential use for enhancing bone regeneration in cranial, oral, maxillo-facial and long fractured bones.

Bone marrow stromal stem cells (BMSCs, also known as mesenchymal stem cells) are a group of adult non-hematopoietic stem cells that are characterized by owing self-renewal capacity, clonogenicity, and differentiation potential into mesoderm-type cells including osteoblast, adipocyte and chondrocyte [5–8]. Several pre-clinical and clinical studies have shown the bone regenerative capacity of adult BMSCs-based therapy for the treatment of several bone loss disease including osteoporosis and oral, cranial, maxillo-facial and long bone defects [9]. Thus, identifying factors that acting directly on BMSCs to promote their differentiation into osteoblast cell lineage, will lead to enhance bone formation.

Coumarin derivatives are naturally plant-derived compounds that can be found in many plants, including, citrus fruits, grasses, and legumes [10]. Coumarins demonstrated a wide variety of pharmacological activities including antibacterial, antifungal, anti-inflammatory, anti-HIV, antioxidant, anticoagulant, and antitumor agents. Due to the biological activities and structure–function relationships, coumarins and their derivatives showed therapeutic potentials for multiple diseases [10–13]**.** In this context, several coumarin derivatives were shown to have bone anabolic effects, including imperatorin and bergapten [14], Daphnetin [15], Osthole [16], Psoralen [17]. In this report, we have studied the effect of newly extracted phytochemical coumarin derivative, 5′-hydroxy auraptene,[7-(5-Hydroxy-3,7-dimethylocta-2,6-dienyloxy)-chromen-2-one], from *Lotus lalambensis* Schweinf on the BMSCs differentiation into osteoblasts. Interestingly, 5′-HA showed to enhance osteoblast differentiation in a dose-dependent manner via BMP2-mediated signaling pathway.

## Materials and methods

### Extraction and purification of 5′-HA

#### Collection of plant material

5′-HA was extracted from *Lotus lalambensis* Schweinf. The roots of *Lotus lalambensis* Schweinf were collected from Eastern province, Al-Hassa, Saudi Arabia. Authenticated at Herbarium of Botany Department, Faculty of Science, Cairo University where voucher specimens have been placed.

### Extraction and purification

The extraction was carried out using petroleum ether at room temperature. After evaporation until dryness, the extract was obtained in yields (w/w) of 5, 55. A sample from petroleum ether extract (15.25 g) was applied to a Sephadex LH-20 column equilibrated with: PE:MeOH:CHCl3 (2,1:1). After comparison with TLC five fractions were obtained 1: (0.4076 g), 2: (1.66 g), 3: (4.25 g), 4: (0.734 g), 5: (0.0813) g. Fraction 3 (4 g) was applied to a Sephadex LH-20 column. After comparison with TLC, fractions were combined resulting in five new fractions: 1: 0.0055 g, 2: 0.0661 g, 3: 0.2234 g, 4: 0.5321 g, 5: 0.7221 g. From fractions, 1–5, pure compound **(**85.66 mg) was obtained. The compound was identified as 7-(5-Hydroxy-3,7-dimethylocta-2,6-dienyloxy)-chromen-2-one also known as 5′-Hydroxy Auraptene depending on NMR data (Additional file 1: Figure S1; Additional file 2: Figure S2) and IR spectra. IR was analyzed in a FTIR Shimadzu Prestige-21 instrument; NMR spectra were assessed using a Bruker Avance 300 in chloroform solutions with standard Bruker software.

### Cell cultures and reagents

Mouse BMSCs were isolated from 8-weeks-old male C57BL/6 J mice as described previously described [18]. Bone marrow was flushed out from mouse tibia and femur into Eppendorf tubes, centrifuged for 1 min at 400 g to collect the marrow cells. Cell were then filtrated through a 70-μm nylon mesh filter and cultured in 175 cm2 flasks in basal culture media (BCM) consists of RPMI-1640 medium supplemented with 12% FBS (Gibco Invitrogen, USA), 12 μM L-glutamine (Invitrogen) and 1% penicillin/streptomycin (P/S) (Gibco Invitrogen, USA). After 24 h, non-adherent cells were removed from the cultured medium, washed with PBS, and cultured in 30 ml of fresh medium in 60 cm2. Medium was changed every 3–4 days and cells were washed, trypsinized and regularly sub-cultured.

Human BMSC cells (hBMSCs) were purchased from Cell Applications Inc. (San Diego, CA). Cells were cultured according to the manufacturer’s instruction, in Dulbecco’s modified Eagle medium (DMEM)/low glucose (Sigma-Aldrich) containing 10% FBS Gibco Invitrogen, USA) and 1% penicillin/streptomycin. Non-adherent cells were removed after 2 days in culture and medium was changed every 2–3 days.

### Cell toxicity assay

The toxicity of 5′-HA was determined by MTT assay to using cell proliferation MTT assay kit according to the manufacturer’s instructions kit (Sigma-Aldrich). Cells were incubated with MTT solution to metabolize to formazan. Absorbance was measured at a wavelength of 550 nm.

### Cell proliferation study

Cell growth was determined in vitro by culturing the cells at 2000 cells/well in 4 well plates in basal culture media (BCM) supplemented with 2% FBS. Cells were trypsinized and counted by the hemocytometer.

### Osteoblast differentiation

Cells were plated at 10 × 10^3^ cells/cm^2^. At 70% confluence, cells were induced to differentiate into osteoblasts osteogenic induction medium (OIM) consists of in α-minimum essential medium (α-MEM; Gibco) supplemented with 10% FBS, 100 U/mL of penicillin, 100 mg/mL of streptomycin, 50 mg/mL of vitamin C (Sigma-Aldrich ApS), and 10 mM β-glycerol-phosphate (Sigma-Aldrich ApS). The medium was changed every third day during the whole differentiation period.

### Adipocyte differentiation

Cells were plated at 15,000 cells/cm2. At 100% confluence, cells were induced in adipogenic-induction medium (AIM) consists of DMEM supplemented with 9% horse serum, 450 μM 1-methyl-3-isobutylxanthine (IBMX), 250 nM dexamethasone, 5 μg/mL insulin (Sigma-Aldrich) and 1 μM rosiglitazone (BRL 49653, Cayman Chemical, Ann Arbor, Michigan). The medium was changed every 3 days during the time course of differentiation.

### Alkaline phosphatase (ALP) activity assay

Cells were cultured in 96 well plate and induced with OIM for 6 days. Cell viability was determined using the CellTiter-Blue® cell viability assay according to the manufacturer’s instructions (Promega, USA) at OD 579. ALP activity was determined by incubating the cells with 1 mg/mL of P-nitro phenyl phosphate in 50 mM NAHCO3 and 1 mM MgCl2 buffer (pH 9.6) at 37 °C for 20 min. The reaction was stopped by the addition of 3 M NaOH. The reaction absorbance was measured at 405 nm and ALP activity was represented after normalization to the cell viability and each sample was measured in 6 replicates.

### Cytochemical staining

#### Alkaline phosphatase staining

Cells were fixed with acetone/citrate buffer pH 4.2 (1.5:1) for 5 min at room temperature and stained with Napthol-AS-TR-phosphate solution (Sigma-Aldrich ApS) for 1 h at room temperature. Napthol-AS-TR-phosphate solution consists of Napthol-AS-TR-phosphate diluted 1:5 in H2O and Fast Red TR (Sigma-Aldrich ApS) diluted 1:1.2 in 0.1 M Tris buffer, pH 9.0, after which both solutions were mixed 1:1.

#### Alizarin red S staining and quantification

Cells were induced to differentiate into osteoblasts for 10–12 days and fixed with 70% ice-cold ethanol for 1 h at − 20 °C. Alizarin red (40 mM; Sigma-Aldrich ApS) dissolved in distilled water, pH = 4 was used to stain the cells for 10 min at room temperature. The quantification of calcium deposition was evaluated by the elution of AR-S with 10% cetylpyridinium chloride (Sigma-Aldrich ApS) for 1 h at room temperature. The absorbance of the eluted dye was measured at 570 nm.

#### Western blot analysis

Cells were treated according to the experiment conditions and then collected at specific time points, washed in cold PBS buffer and lysed in cell lysis buffer (10 mM Tris–HCl, pH 7.4, 150 mM sodium chloride, 1% NP-40, 0.1% SDS, 1 mM EDTA, 1 mM phenyl-methylsulfonyl fluoride, 1 mM NaF, 1 mM Na3VO4), supplemented with protease inhibitor cocktail (Roche Diagnostics, Mannheim, Germany). 20 μg of protein was separated on 8–12% NuPAGE® Novex® Bis-Tris gel systems (Thermo Fisher Scientific, Germany) followed by transfer to PVDF membrane (Millipore, USA). The membrane was blocked and incubated with peroxidase-conjugated secondary antibody (Santa Cruz Biotechnology, Heidelberg Germany). Proteins were visualized by ECL chemiluminescence (Thermo Fisher Scientific, Roskilde, Germany). Antibodies for Smad1/5/8 (total or phosphor) were obtained from Santa Cruz Biotechnology, Inc. (Heidelberg, Germany). Quantification of western blots was performed with ImageJ program.

#### RNA extraction and real-time PCR analysis

RNA was extracted from cultured cells using a single-step method of TRIzol (Thermo Fisher Scientific, Roskilde, Germany). cDNA was synthesized from 1 μg of total RNA using revertAid H minus first strand cDNA synthesis kit (Fermentas, St Leon-Rot, Germany) according to the manufacturer’s instructions. Quantitative real time PCR was performed with Applied Biosystems 7500 Real-Time system using Fast SYBR® Green Master Mix (Applied Biosystems, California, USA) with specific primers (Additional file 4: Table S1). The expression of each target gene was normalization to β-Actin and Hprt mRNA expression as reference genes, using a comparative CT method [(1/ (2delta-CT) formula, where delta-CT is the difference between CT-target and CT-reference] with Microsoft Excel 2007® as described [19].

#### PCR Array analysis

RNA was extracted and cDNA was synthesized as described above. RT^2^ Profiler™ PCR array mouse osteogenesis with 84 genes associated with osteogenic induction (Qiagen GmbH, Germany) was performed for each sample in triplicates using SYBR® Green quantitative PCR method on Applied Biosystems 7500 real-time PCR system and data were analyzed according to the manufacturer’s instructions.

#### Statistical analysis

All values are expressed as mean ± SD (standard deviation), of at least 3 independent experiments. Power calculation was performed for 2-samples using unpaired Student’s T-test (2-tailed) assuming equal variation in the two groups. Differences were considered statistically significant at *P < 0.05, and **P < 0.005.

## Results

### 5′-HA does not affect cell proliferation of mBMSCs

**Fig. 1:**
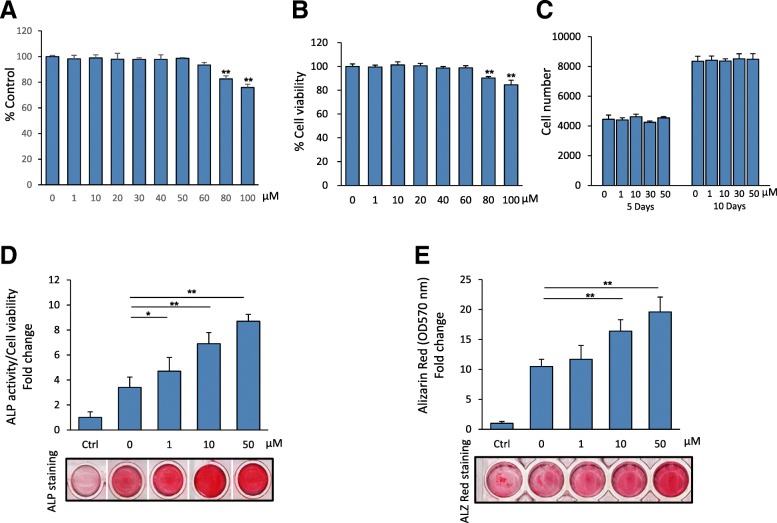
5′-HA promotes the differentiation of mBMSCs into osteogenic cell lineage. Cytotoxicity of 5′-HA on cultured primary mBMSCs as measured by MMT assay (**a**) and Cell Titer-Blue® cell viability assay (**b**) after 3 days in culture. **c** Effect of 5′-HA (1–50 μM) on cell proliferation of cultured mBMSCs as measured by counting the cell number at different concentrations of 5′-HA after 5 and 10 days. **d** Dose-dependent stimulatory effect of 5′-HA on osteoblast differentiation of mBMSCs as measured by quantification of ALP activity after 7 days of induction and (**e**) quantitative Alizarin red staining for matrix mineralization after 12 days of induction. Cells were either cultured without osteogenic induction media (Ctrl, control), or induced to osteogenic lineage in the absence (0) or the presence of different concentrations of 5′-HA. Values were normalized to cell viability and represented as fold change over control non-induced cells. Stained images were shown for both ALP activity and Alizarin red staining. Values are mean ± SD of three independent experiments, (*p < 0.05, **p < 0.005 compared to control without 5′-HA for A&B; and compared to induced cells without 5′-HA for **c**&**d**)

We studied the cytotoxicity of 5′-HA by culturing mBMSCs in the presence of different concentration of 5′-HA (1–100 μM) for 3 days and measure cell viability by MTT assay and Cell Titer-Blue. The results of both assays showed that the toxic effect of 5′-HA on cell viability started at the concentration above 60 μM (Fig. 1a&b). Thus, we selected the concentrations between 10 and 50 μM to be used in all experiments of this study. We then showed that the treatment of mBMSCs with 5′-HA at concentrations range from 1 to 50 μM did not exert any effect on their cell proliferation (Fig. 1c) after 5 and 10 days of treatment in culture as assessed by counting of cell number.

### 5′-HA stimulates osteoblast differentiation of mBMSCs

**Fig. 2:**
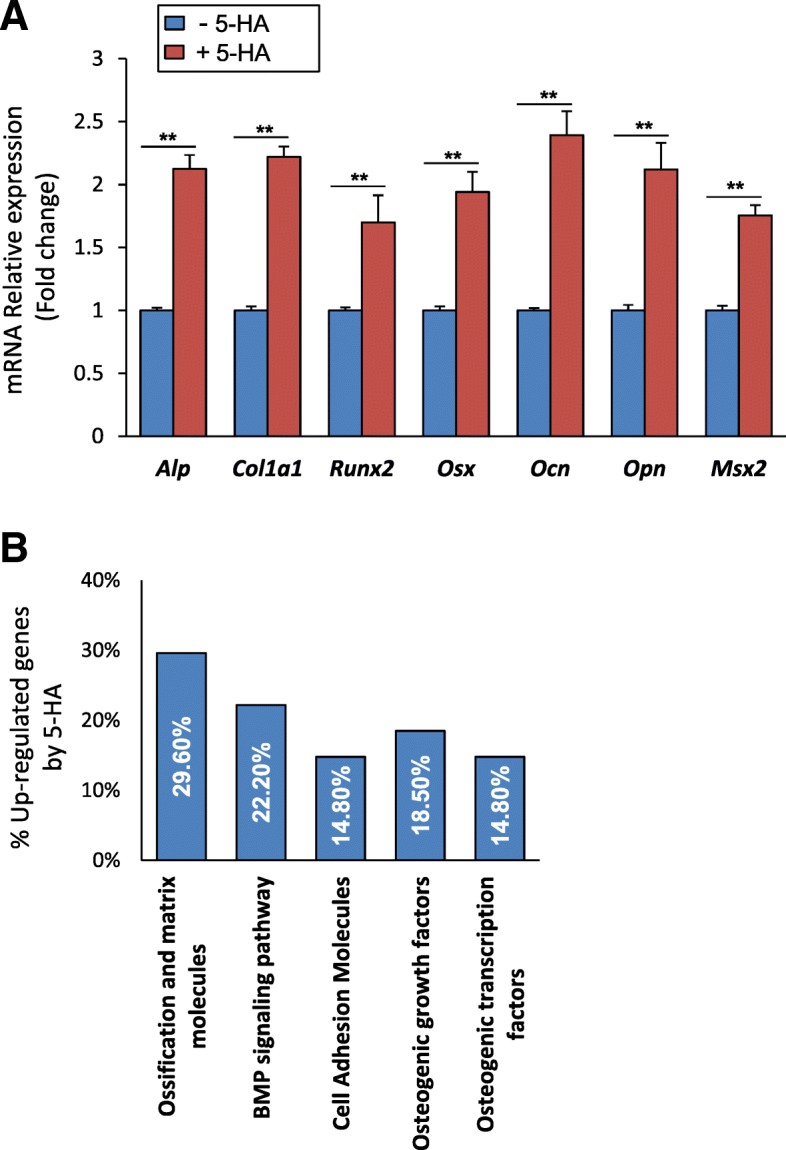
5′-HA stimulates the mRNA expression of osteogenic markers in mBMSCs. **a** QPCR analysis of osteogenic markers expression in mBMSCs that induced to osteoblast differentiation without (control) or with 50 μM 5′-HA for 7 days. Each target gene was normalized to reference genes as described in M&M and genes were represented as fold changes over induced cells without 5′-HA. **b** Upregulation of different osteogenic-related genes in mBMSCs by 5′-HA (50 μM) as measured by osteogenic RT2 profiler array as described in M&M. Cells were induced to osteogenic lineage in absence or presence of 5′-HA for 7 days. Data were represented as percentage of categorized genes according to Table 1. Values are mean ± SD of three independent experiments, (*p < 0.05, **p < 0.005)

We examined the effect of 5′-HA on the differentiation of mBMSCs into osteoblastic cell lineage. As shown in Fig. 1d&e, 5′-HA promoted the differentiation of mBMSCs into osteoblasts in a dose-dependent manner as measured by significant increased ALP activity and Alizarin Red staining for matrix mineralization. In consistent, 5′-HA increased the mRNA expression of early (*Alp, Col1a1, Msx2 and Runx2*) and late (*Ocn* and *Opn*) osteogenic markers (Fig. 2a). In addition, 5′-HA significantly up-regulated 64.3% (≥2 fold, *p* < 0.05) of the differentially expressed osteoblastic genes during osteogenesis of mBMSCs versus control cells without 5′-HA treatment as assessed by real time PCR-based osteogenic gene array analysis (Fig. 2b, Table 1). Interestingly, 22.2% of the up-regulated genes by 5′-HA was related to the activation of BMP-signaling pathway (Fig. 2b). These data clearly demonstrated the stimulatory effect of 5′-HA on osteogenesis of mBMSCs.

**Table 1:** Up-regulation of osteogenic genes expression in mBMSCs treated with 50 μM of 5′-HA for 6 days during osteoblast differentiation

Gene name	Gene Symbol	Fold change
Ossification and matrix molecules		
Alkaline phosphatase, liver/bone/kidney	*Alpl*	8.3
Bone gamma carboxyglutamate protein	*Bglap*	6.2
Biglycan	*Bgn*	4.5
Collagen type I alpha 1	*Col1a1*	3.2
Collagen type I alpha 2	*Col1a2*	2.3
Collagen type V alpha 1	*Col5a1*	2.1
FMS-like tyrosine kinase 1	*Flt1*	
Secreted phosphoprotein 1 (Osteopontin)	*Spp1*	5.3
BMP signaling pathway		
Bone morphogenetic protein 2	*Bmp2*	7.5
Bone morphohenitic protein 4	*Bmp4*	4.2
Bone morphogenetic protein 7	*Bmp7*	2.7
Bone morphogenetic protein receptor. Type 1A	*Bmpr1a*	2.1
Bone morphohenitic protein receptor, type 1B	Bmpr1a	3.4
MAD homolog 5 (Drosophila)	*Smad5*	2.8
Cell Adhesion Molecules		
Fibronectin 1	*Fn1*	4.3
Integrin beta 1 (fibronectin receptor beta)	*Itgb1*	3.2
Integrin alpha 2	*Itga2*	4.2
Integrin alpha 2b	*Itga2b*	2.9
Osteogenic growth factors		
Fibroblast growth factor receptor 2	*Fgfr2*	2.8
Insulin-like growth factor 1	*Igf1*	8.4
Insulin-like growth factor I receptor	*Igf1r*	4.3
Platelet derived growth factor. Alpha	*Pdgfa*	3.8
Vascular endothelial growth factor A	*Vegfa*	4.2
Osteogenic transcription factors		
Distal-less homeobox 5	*Dlx5*	6.4
Runt related transcription factor 2	*Runx2*	5.9
Sp7 transcription factor 7	*Sp7*	4.6
Twist gene homolog 1	*Twist1*	4.3

Cells were induced to differentiate into osteoblast without (control) or with 50 μM 5′-HA for 6 days. Mouse osteogenesis RT^2^ Profiler™ PCR array with 84 osteoblast genes was performed for each cDNA sample using the SYBR® Green quantitative PCR method. Up-regulated genes by BMSCs in the presence of 5′-HA were represented as fold change over control differentiated cells without 5’-HA

### 5′-HA exerts no effect on the differentiation of mBMSCs into adipocytes

**Fig. 3:**
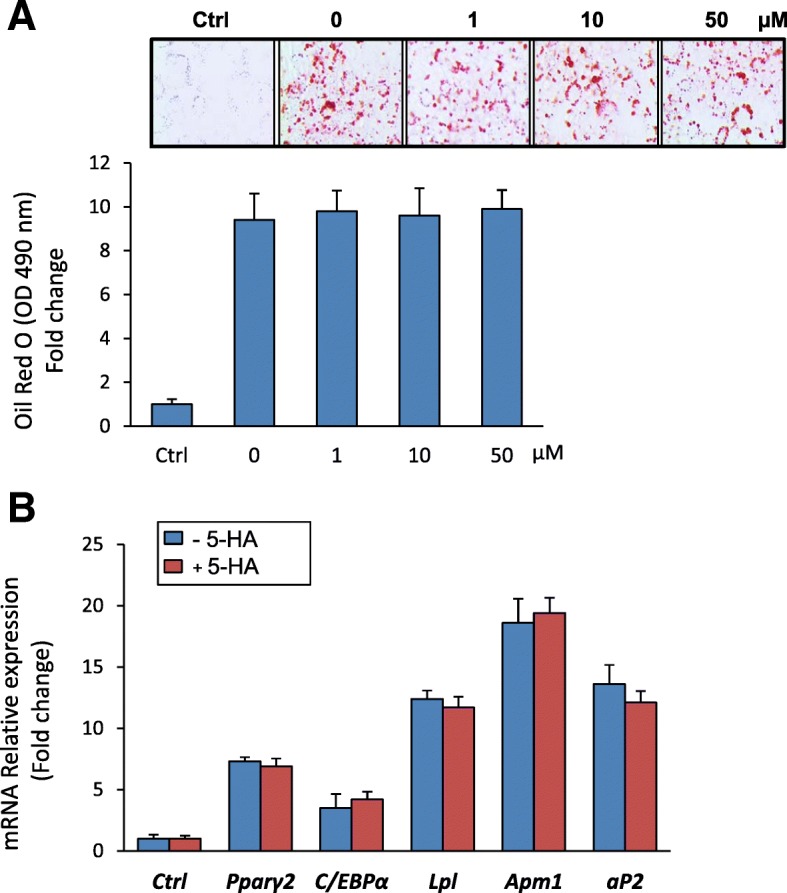
5′-HA does not affect the adipocyte differentiation of mBMSCs. **a** 5′-HA does not affect adipocyte differentiation of mBMSCs as measured by Oil red O staining and its quantification. Cells were either cultured without adipogenic induction media (Ctrl, control), or induced to adipogenic lineage in the absence (0) or the presence of different concentrations of 5′-HA. Images of Oil red O staining for fat droplets accumulation in differentiated mBMSCs were shown. **b** QPCR analysis of mRNA expression of adipogenic markers at day 12 of the differentiated mBMSCs into adipocyte with or without 5′-HA (50 μM). Each target gene was normalized to reference genes and represented as fold change over induced control (0). Values are mean ± SD of three independent experiments, (*p < 0.05, **p < 0.005)

Since osteoblasts and adipocytes in bone marrow are derived from the same mBMSCs [6], we studied the effect of 5′-HA on the differentiation of mBMSCs into adipocytes. 5′-HA at different concentrations did not affect the adipogenesis of mBMSCs as revealed by quantification of Oil Red O staining for lipid accumulation (Fig. 3a). In addition, treatment of mBMSCs with 5′-HA significantly did not show any effect on the transcriptional expression of either early (*Pparγ2and C/ebpα*) or late (*aP2, Apm1, Lpl*) adipogenic markers compared to non-treated cells as assessed by real time PCR analysis (Fig. 3b).

### 5′-HA stimulates BMP2-induced osteoblast differentiation of mBMSCs

**Fig. 4:**
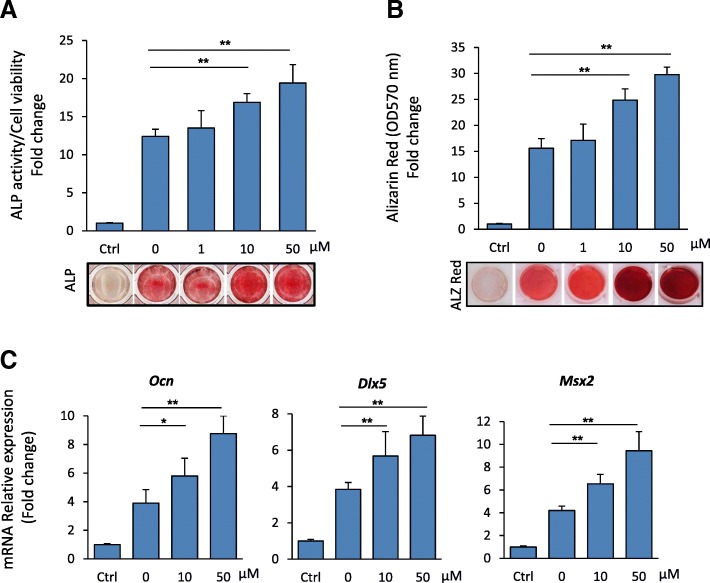
5′-HA stimulates BMP-induced osteoblast differentiation. **a** Dose-dependent stimulatory effect of 5′-HA on BMP2-induced osteoblast differentiation of mBMSCs as assessed by quantification of ALP activity after 7 days of induction and (**b**) quantitative Alizarin red staining for matrix mineralization after 12 days of induction. Cells were induced to osteoblast differentiation using BMP2 (50 ng/ml) in the absence (0) or the presence of different concentrations of 5′-HA. Representative images were shown for Alizarin Red staining. **c** 5′-HA stimulates the expression of BMP2-related osteogenic markers in a dose-dependent manner as measured by qPCR analysis. Each target gene was normalized to reference genes and represented as fold change over induced control (0) without 5′-HA. Values are mean ± SD of three independent experiments, (*p < 0.05, **p < 0.005 compared to control induced cells without 5′-HA)

Based on our finding that, genes related to BMP2-signaling pathway were upregulated by 5′-HA during osteogenesis (Fig. 2b), we studied the effect of 5′-HA on BMP2-induced osteogenesis in mBMSCs. Thus, cells were induced with BMP2 to differentiate into osteoblast in the absence or the presence of different concentrations of 5′-HA. As shown in Fig. 4a&b, 5′-HA showed to stimulate BMP2-induced ALP activity and matrix mineralization in dose-dependent manner as compared to BMP2-treated cells without 5′-HA. In addition, real time PCR analysis revealed the dose-dependent stimulatory effect of 5′-HA on the expression of downstream targets of BMPs signaling (*Ocn, Dlk5* and *Msx2*) as assessed by qPCR (Fig. 4c).

### 5′-HA stimulates osteoblast differentiation of mBMSCs in BMP signaling dependent mechanism via upregulating SMAD4 expression

**Fig. 5:**
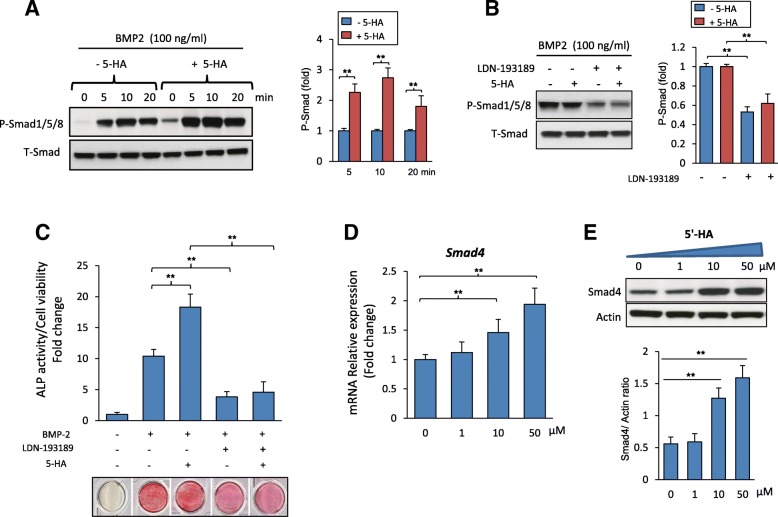
5′-HA stimulates the differentiation of mBMSCs into osteobalst via activating BMP signaling pathway. **a** Western blot analysis of stimulating the phosphorylation of Smad1/5/8 in mBMSCs by 5′-HA (50 μM) versus control. **b** Western blot analysis of inhibiting BMP signaling pathway by specific BMP1R inhibitor, (LDN-193189, 10 μM) in absence and presence of 5′-HA. **c** Inhibitory effect of BMP1R inhibitor (LDN-193189, 10 μM) on the stimulatory effect of 5′-HA (50 μM) on BMP2-induced osteogenesis in mBMSCs as measured by quantitative ALP activity. **d** Dose-dependent stimulatory effect of 5′-HA on *Smad4* mRNA expression and (**e**) SMAD4 protein expression in BMP2-treated mBMSCs as measured by qPCR and Western blot analysis. Cells were induced with BMP2 in the absence (0) or the presence of 5′-HA (different concentrations) for 24 h. Each target gene was normalized to reference genes and represented as fold change over induced control (0) without 5′-HA. Values are mean ± SD of three independent experiments, (**p < 0.005)

To examine whether BMP-signaling is involved in mediating the stimulatory effect of 5′-HA on osteogenesis in mBMSCs, we further examined the effect of 5′-HA on the activation of BMP2 signaling in mBMSCs. Treatment of mBMSCs with 5′-HA displayed significant activation of Smad 1/5/8 phosphorylation as shown by Western blot analysis (Fig. 5a). In support to this finding, inhibition of the BMP2 signaling using LDN-193189, a specific BMP1R inhibitor, showed to abolish the stimulatory effect of 5′-HA on Smad1/5/8 phosphorylation (Fig. 5b). Interestingly, treatment of mBMSCs with LDN-193189 inhibitor in the presence of 5′-HA showed to significantly attenuate the stimulatory effect of 5′-HA on osteogenesis of mBMSCs as assessed by reduced ALP activity with 74.8% as compared to mBMSCs without LDN-193189 (Fig. 5c).

To determine the mechanism underlying the stimulatory effect of 5′-HA on BMP signaling, we studied the effect of 5′-HA on the mRNA expression of several BMPs antagonists and *Smad4* as a crucial downstream molecule of BMP signaling. 5′-HA did not exert any effect on the transcription of BMPs antagonists including SMAD1/5 E3 ubiquitin protein ligase, *Smurf1/2*, Noggin, Gremlin1/2, Chordin as revealed by qPCR analysis of gene expression (Additional file 3: Figure S3 A). Interestingly, treatment of mBMSCs with 5′-HA in the presence of BMP2 showed to increase *Smad4* mRNA expression by 110% as compared to non-treated cells (Additional file 3: Figure S3 B). In addition, 5′-HA upregulated *Smad4* expression at both mRNA and protein levels in dose-dependent manner as assessed by qPCR and Western blot analysis in dose dependent-manner (Fig. 5d&e).

### 5′-HA promotes BMP2-induced osteoblast differentiation of human BMSCs

**Fig. 6:**
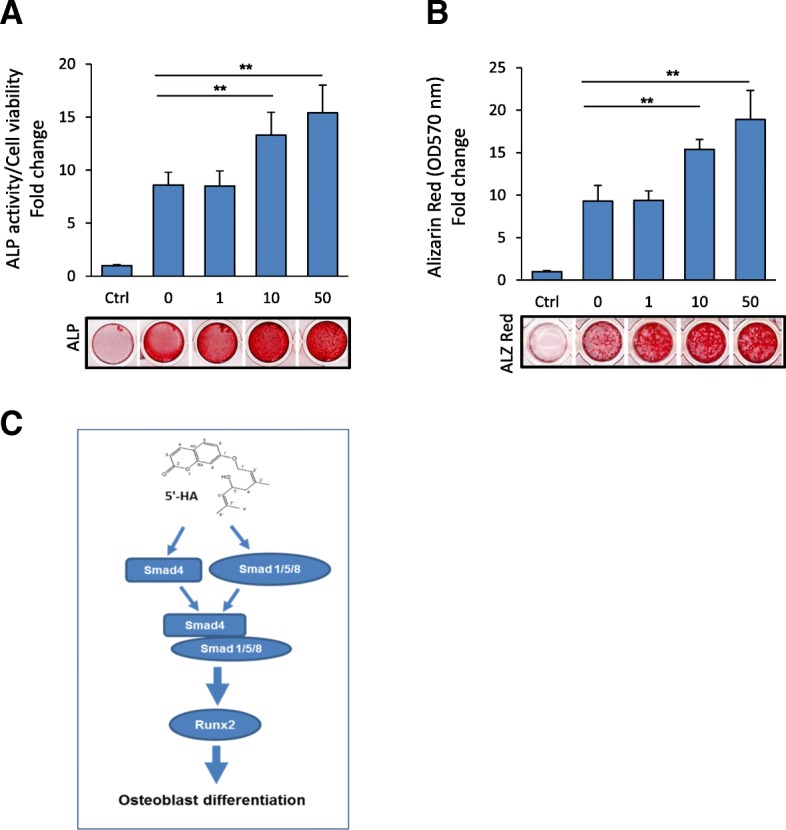
5′-HA promotes BMP-induced osteoblast differentiation of hBMSCs. **a** Dose-dependent stimulatory effect of 5′-HA on BMP2-induced osteoblast differentiation in hBMSCs as assessed by quantification of ALP activity and (**b**) quantitative Alizarin red staining for matrix mineralization. Cells were induced to osteoblast differentiation using BMP2 (100 ng/ml) in the absence (0) or the presence of different concentrations of 5′-HA for either 7 days (ALP activity) or 12 days (Alizarin Red staining). Representative images of ALP and Alizarin red staining were shown under each corresponding graph. **c** Mode of action of 5′-HA on promoting osteogenesis in BMP-signaling dependent mechanism. Values are mean ± SD of three independent experiments, (*p < 0.05, **p < 0.005 compared to BMP2-treated cells without 5′-HA)

We further examined the effect of 5′-HA on promoting BMP2-induced osteogenesis in human (h) BMSCs. Interestingly, 5′-HA showed to promote BMP2-induced osteoblast differentiation of hBMSCs in dose-dependent manner as assessed by significant stimulation of ALP activity and Alizarin red staining for matrix mineralization compared to non-treated cells with 5′-HA (Fig. 6 a&b).

## Discussion

This is the first report to study the effect of 5′-HA, a coumarin derivative newly isolated from *Lotus lalambensis* Schweinf on the differentiation of mBMSCs into osteoblasts and adipocytes. Our data demonstrated the specific stimulatory effect of 5′-HA on the differentiation of mBMSCs into osteogenic cell lineage without affecting their differentiation into adipocytic lineage. In addition, 5′-HA-induced osteogenesis was found to be mediated in BMP signaling-dependent mechanism.

Our data demonstrated that 5′-HA stimulated ALP activity and matrix mineralization of mBMSCs in a dose-dependent manner. This finding was supported by showing that the treatment of mBMSCs with 5′-HA significantly up-regulated the two main osteogenic transcription factors *Runx2* and *Msx2* and other related osteogenic markers. In consistent, several coumarin derivatives were reported to exert stimulatory effect on osteogenesis of osteoblastic cells as well as BMSCs. These include, Imperatorin and Bergapten, two furanocoumarins, which showed to enhance the osteogenesis of primary rat osteoblasts [14]. Osthole, a naturally-derived coumarin was reported to promote osteoblast differentiation of primary mouse calvarial pre-osteoblast cells, MC3T3-E1 cell line and osteoblast-like UMR106 cells [16, 20, 21] and Psoralen, extracted from Chinese herbs was reported to stimulate osteogenesis of primary mouse calvarial osteoblasts in dose-dependent manner [22]. Furthermore, recently, Isopsoralen, a natural compound belongs to angular furanocoumarins was reported to stimulate the differentiation of BMSCs into osteoblasts [23].

In our study, 5′-HA did not exert any action on the differentiation of mBMSCs into adipocytes, as revealed by the absence of any effects on either lipid accumulation measured by Oil red O staining or gene expression of early and late adipogenic markers. This finding suggests the specificity of 5′-HA in directing the differentiation fate of mBMSCs into osteoblastic lineage only without affecting their differentiation into adipocytic lineage. In this context, the effect of coumarin derivatives on adipogenesis was found to be contradictory in literatures. Several reports demonstrated the function of coumarin derivatives as ligand for PPARs, a ligand-activated transcription factors that regulate lipid metabolism [24]. For examples, Osthole and citrus Auraptene showed to induced adipogenesis in association with activating both PPARα and PPARγ in a dose-dependent manner [25, 26]. Further, Aculeatin was reported to stimulate both adipogenesis and lipolysis of 3T3L1 cell line [27]. On the other hand, other reports showed the ability of coumarin derivatives including Pteryxin, Scoparone, and esculetin to suppress the adipocyte differentiation of 3T3L1 via antagonizing PPARγ-dependent pathway [28–30]. In addition, Bergamottin showed to inhibit adipogenesis in vitro (in 3 T3-L1 cell line) and in vivo (in high fat mice model) [31] and Isopsoralen was reported to attenuate the adipogenesis of BMSCs in vitro and in vivo [23].

Our data, demonstrated the stimulatory effect of 5′-HA on BMP-induced osteogenesis via activation of Smad1/5/8 phosphorylation and increased expression of its downstream osteogenic target genes *Msx2, Osn* and *Dlx5* in a dose-dependent manner. Moreover, blocking of BMP signaling by specific BMP1R inhibitor, significantly abolished the stimulatory effect of 5′- HA on osteogenesis of mBMSCs. BMPs play a pivotal role in bone formation and remodeling. Binding of BMPs to its receptors resulted in activation of Smad1/5/8 phosphorylation which in turn binds to SMAD4 and translocates into the nucleus to up-regulate the expression of bone-related genes including *Col1a1, Alp, Oc, Dlx5* and *Msx2* [32–34]. In this context, we showed that the regulatory effect of 5′-HA on BMP-induced osteogenesis is mediated via upregulating *Smad4* expression. Several studies demonstrated the crucial role of SMAD4 in bone development and homeostasis by mediating both BMPs and TGF-βs signals. Deletion of *Smad4* in osteoblasts showed to inhibit BMP-induced osteogenesis in vitro [35] and to decrease osteoblast proliferation/differentiation and reduce bone mass in vivo [36, 37]. In consistent with our finding, two other coumarin derivatives were reported to mediate its biological function by regulating SMAD4 expression. For example, Osthole, showed to inhibit collagen I and III expressions in TGF-β1 treated mouse cardiac fibroblasts (CFs) by modulating *Smad4* expression [38] and Bergapten was reported to deplete estrogen receptor in breast cancer cells in respond to TGF β 1 by regulating *Smad4* expression [39].

Interestingly, BMP signaling was reported to mediate the stimulatory effect of some coumarin derivatives on osteogenesis. These include, Psoralen and Osthole, that induced osteoblast differentiation in BMP-2 and BMP-4 dependent mechanism [22] and in canonical β-catenin/BMP2-dependent mechanism [40] respectively.

On the other hand, other signaling pathways have also been reported to mediate the regulatory mechanism of coumarin derivatives-induced osteogenesis. For examples, activation of cAMP response element-binding protein signaling [16], and p38 and ERK-dependent signaling [14] were reported to mediate the effect of Osthole and imperatorin on osteogenesis respectively. Thus, it is plausible that other signaling mechanisms could also contributed in mediating the stimulatory effect of 5′-HA on osteogenesis.

## In conclusion

Our study identified 5′-HA, naturally-derived coumarin derivative as a novel osteoanabolic compound that function specifically to promote the differentiation of mBMSCs into osteoblasts without affecting their differentiation into adipocytes. The stimulatory effect of 5′-HA on osteogenesis was found to be mediated in BMP-signaling dependent mechanism via activating the Smad1/5/8 phosphorylation and upregulating the *Smad4* expression (Fig. 6c). Finally, blocking of BMP signaling pathway showed to attenuate 5′-HA-induced osteogenesis. Thus, our data provide 5′-HA as a promising drug for treatment of osteoporosis.

## Additional files

Additional file 1: **Figure S1.** NMR spectrum of 5′-hydroxy-aurapten. (A) 1H NMR spectrum of 5′-hydroxy-aurapten (400 MHz, CDCl3). (B) ^1^H and ^13^C NMR spectral data of5′-hydroxy-aurapten in CDCl3. (PDF 316 kb)

Additional file 2: **Figure S2.** Chemical Structure of 5′-Hydroxy Auraptene. Chemical structure of isolated and purified 7-(5-Hydroxy-3,7-dimethylocta-2,6-dienyloxy)-chromen-2-one. (PDF 201 kb)

Additional file 3: **Figure S2.** Effect of 5′-HA on gene expression of BMPs antagonists and Smad4. (A) 5′-HA did not affect the mRNA expression of BMPs antagonists including *Smurf1/2*, *Nog*, *Gremlin1/2* and *Chrd*, while (B) stimulating the transcription of *Smad4* gene as measured by qPCR analysis. (PDF 467 kb)

Additional file 4: **Table S1.** List of primers used for qPCR. List of primer sequences used for qPCR. (PDF 244 kb)

## Data Availability

All materials are available by the corresponding author.

## Abbreviations

5′-HA: 5-hydroxy aurapten
AIM: Adipogenic-induction medium
ALP: Alkaline phosphatase
aP2: Adipocyte lipid-binding protein 2
APM1: Adiponectin
BCM: Basal culture media
BMP: Bone morphogenetic protein
C/EBP-α: CCAAT/enhancer-binding protein alpha
Col1a1: Collagen type 1
Dlx5: Distal-Less Homeobox 5
LPL: Lipoprotein lipase
mBMSCs: Mouse bone marrow-derived mesenchymal (skeletal) stem cells
MSX2: Homeobox Protein MSX-2
OCN: Osteocalcin
OIM: Osteogenic-induction medium
OPN: Osteopontin
PPARγ2: Peroxisome proliferator-activated receptor gamma 2
QPCR: Quantitative real-time PCR
Runx2: Runt-related transcription factor 2

## References

[1] Lupsa BC, Insogna K (2015). Bone health and osteoporosis. Endocrinol Metab Clin N Am.

[2] Cummings SR, Martin JS, McClung MR, Siris ES, Eastell R, Reid IR, Delmas P, Zoog HB, Austin M, Wang A (2009). Denosumab for prevention of fractures in postmenopausal women with osteoporosis. N Engl J Med.

[3] Valverde P (2008). Pharmacotherapies to manage bone loss-associated diseases: a quest for the perfect benefit-to-risk ratio. CurrMedChem.

[4] Weinerman S, Usera GL (2015). Antiresorptive therapies for osteoporosis. Oral Maxillofac Surg Clin North Am.

[5] Abdallah BM, Ditzel N, Kassem M (2008). Assessment of bone formation capacity using in vivo transplantation assays: procedure and tissue analysis. Methods Mol Biol.

[6] Abdallah BM, Jafari A, Zaher W, Qiu W, Kassem M (2015). Skeletal (stromal) stem cells: an update on intracellular signaling pathways controlling osteoblast differentiation. Bone.

[7] Abdallah BM, Kassem M (2009). The use of mesenchymal (skeletal) stem cells for treatment of degenerative diseases: current status and future perspectives. JCell Physiol.

[8] Bianco P, Robey PG (2015). Skeletal stem cells. Development.

[9] Arvidson K, Abdallah BM, Applegate LA, Baldini N, Cenni E, Gomez-Barrena E, Granchi D, Kassem M, Konttinen YT, Mustafa K (2011). Bone regeneration and stem cells. J Cell Mol Med.

[10] Zhu JJ, Jiang JG. Pharmacological and Nutritional Effects of Natural Coumarins and Their Structure-Activity Relationships. Mol Nutr Food Res. 2018:e1701073. [Epub ahead of print]. 10.1002/mnfr.201701073 29750855

[11] de Souza SM, Delle Monache F, Smania A (2005). Antibacterial activity of coumarins. Zeitschrift fur Naturforschung C, Journal of biosciences.

[12] Detsi A, Kontogiorgis C, Hadjipavlou-Litina D (2017). Coumarin derivatives: an updated patent review (2015-2016). Expert opinion on therapeutic patents.

[13] Kontogiorgis C, Detsi A, Hadjipavlou-Litina D (2012). Coumarin-based drugs: a patent review (2008 -- present). Expert opinion on therapeutic patents.

[14] Tang CH, Yang RS, Chien MY, Chen CC, Fu WM (2008). Enhancement of bone morphogenetic protein-2 expression and bone formation by coumarin derivatives via p38 and ERK-dependent pathway in osteoblasts. Eur J Pharmacol.

[15] Liu Xifang, Gao Xiaohang, Liu Yuanxin, Liang Dongsheng, Fu Ting, Song Yixin, Zhao Congzhe, Dong Bo, Han Weihua (2018). Daphnetin inhibits RANKL‐induced osteoclastogenesis in vitro. Journal of Cellular Biochemistry.

[16] Zhang Zhong-Rong, Leung Wing, Li Gang, Kong Siu, Lu Xiong, Wong Yin, Chan Chun (2017). Osthole Enhances Osteogenesis in Osteoblasts by Elevating Transcription Factor Osterix via cAMP/CREB Signaling In Vitro and In Vivo. Nutrients.

[17] Wong RW, Rabie AB (2011). Effect of psoralen on bone formation. J Orthop Res.

[18] Peister A, Mellad JA, Larson BL, Hall BM, Gibson LF, Prockop DJ (2004). Adult stem cells from bone marrow (MSCs) isolated from different strains of inbred mice vary in surface epitopes, rates of proliferation, and differentiation potential. Blood.

[19] Abdallah BM (2017). Marrow adipocytes inhibit the differentiation of mesenchymal stem cells into osteoblasts via suppressing BMP-signaling. J Biomed Sci.

[20] Meng F, Xiong Z, Sun Y, Li F (2004). Coumarins from Cnidium monnieri (L.) and their proliferation stimulating activity on osteoblast-like UMR106 cells. Die Pharmazie.

[21] Zhang Q, Qin L, He W, Van Puyvelde L, Maes D, Adams A, Zheng H, De Kimpe N (2007). Coumarins from Cnidium monnieri and their antiosteoporotic activity. Planta Med.

[22] Tang DZ, Yang F, Yang Z, Huang J, Shi Q, Chen D, Wang YJ (2011). Psoralen stimulates osteoblast differentiation through activation of BMP signaling. Biochem Biophys Res Commun.

[23] Wang J, Li SF, Wang T, Sun CH, Wang L, Huang MJ, Chen J, Zheng SW, Wang N, Zhang YJ (2017). Isopsoralen-mediated suppression of bone marrow adiposity and attenuation of the adipogenic commitment of bone marrow-derived mesenchymal stem cells. Int J Mol Med.

[24] Corrales Patricia, Vidal-Puig Antonio, Medina-Gómez Gema (2018). PPARs and Metabolic Disorders Associated with Challenged Adipose Tissue Plasticity. International Journal of Molecular Sciences.

[25] Kuroyanagi K, Kang MS, Goto T, Hirai S, Ohyama K, Kusudo T, Yu R, Yano M, Sasaki T, Takahashi N (2008). Citrus auraptene acts as an agonist for PPARs and enhances adiponectin production and MCP-1 reduction in 3T3-L1 adipocytes. Biochem Biophys Res Commun.

[26] Liang HJ, Suk FM, Wang CK, Hung LF, Liu DZ, Chen NQ, Chen YC, Chang CC, Liang YC (2009). Osthole, a potential antidiabetic agent, alleviates hyperglycemia in db/db mice. Chem Biol Interact.

[27] Watanabe A, Kato T, Ito Y, Yoshida I, Harada T, Mishima T, Fujita K, Watai M, Nakagawa K, Miyazawa T (2014). Aculeatin, a coumarin derived from Toddalia asiatica (L.) lam., enhances differentiation and lipolysis of 3T3-L1 adipocytes. Biochem Biophys Res Commun.

[28] Noh JR, Kim YH, Hwang JH, Gang GT, Yeo SH, Kim KS, Oh WK, Ly SY, Lee IK, Lee CH (2013). Scoparone inhibits adipocyte differentiation through down-regulation of peroxisome proliferators-activated receptor gamma in 3T3-L1 preadipocytes. Food Chem.

[29] Nugara RN, Inafuku M, Takara K, Iwasaki H, Oku H (2014). Pteryxin: a coumarin in Peucedanum japonicum Thunb leaves exerts antiobesity activity through modulation of adipogenic gene network. Nutrition (Burbank, Los Angeles County, Calif).

[30] Shin E, Choi KM, Yoo HS, Lee CK, Hwang BY, Lee MK (2010). Inhibitory effects of coumarins from the stem barks of Fraxinus rhynchophylla on adipocyte differentiation in 3T3-L1 cells. Biol Pharm Bull.

[31] Ko JH, Nam D, Um JY, Jung SH, Ahn KS (2018). Bergamottin inhibits Adipogenesis in 3T3-L1 cells and weight regulation in diet-induced obese mice. The American journal of Chinese medicine.

[32] Afzal F, Pratap J, Ito K, Ito Y, Stein JL, van Wijnen AJ, Stein GS, Lian JB, Javed A (2005). Smad function and intranuclear targeting share a Runx2 motif required for osteogenic lineage induction and BMP2 responsive transcription. J Cell Physiol.

[33] Franceschi RT, Xiao G (2003). Regulation of the osteoblast-specific transcription factor, Runx2: responsiveness to multiple signal transduction pathways. J Cell Biochem.

[34] Wu M, Chen G, Li YP (2016). TGF-beta and BMP signaling in osteoblast, skeletal development, and bone formation, homeostasis and disease. Bone research.

[35] Nojima J, Kanomata K, Takada Y, Fukuda T, Kokabu S, Ohte S, Takada T, Tsukui T, Yamamoto TS, Sasanuma H (2010). Dual roles of smad proteins in the conversion from myoblasts to osteoblastic cells by bone morphogenetic proteins. J Biol Chem.

[36] Salazar VS, Zarkadis N, Huang L, Norris J, Grimston SK, Mbalaviele G, Civitelli R (2013). Embryonic ablation of osteoblast Smad4 interrupts matrix synthesis in response to canonical Wnt signaling and causes an osteogenesis-imperfecta-like phenotype. J Cell Sci.

[37] Tan X, Weng T, Zhang J, Wang J, Li W, Wan H, Lan Y, Cheng X, Hou N, Liu H (2007). Smad4 is required for maintaining normal murine postnatal bone homeostasis. J Cell Sci.

[38] Liu JC, Wang F, Xie ML, Cheng ZQ, Qin Q, Chen L, Chen R (2017). Osthole inhibits the expressions of collagen I and III through Smad signaling pathway after treatment with TGF-beta1 in mouse cardiac fibroblasts. Int J Cardiol.

[39] Panno ML, Giordano F, Rizza P, Pellegrino M, Zito D, Giordano C, Mauro L, Catalano S, Aquila S, Sisci D (2012). Bergapten induces ER depletion in breast cancer cells through SMAD4-mediated ubiquitination. Breast Cancer Res Treat.

[40] Tang DZ, Hou W, Zhou Q, Zhang M, Holz J, Sheu TJ, Li TF, Cheng SD, Shi Q, Harris SE (2010). Osthole stimulates osteoblast differentiation and bone formation by activation of beta-catenin-BMP signaling. J Bone Miner Res.

