# Supplementary material for: 5′-hydroxy Auraptene stimulates osteoblast differentiation of bone marrow-derived mesenchymal stem cells via a BMP-dependent mechanism
Source: J Biomed Sci. 2019 Jul 5;26:51. doi: 10.1186/s12929-019-0544-7 (PMC6610929; doi:10.1186/s12929-019-0544-7)
Supplement: Supplementary file 1 — Figure S1. NMR spectrum of 5′-hydroxy-aurapten. (A) 1H NMR spectrum of 5′-hydroxy-aurapten (400 MHz, CDCl3). (B) 1H and 13C NMR spectral data of5′-hydroxy-aurapten in CDCl3. (PDF 316 kb) [file 12929_2019_544_MOESM1_ESM.pdf]

Additional file 1: Figure S1

(A)

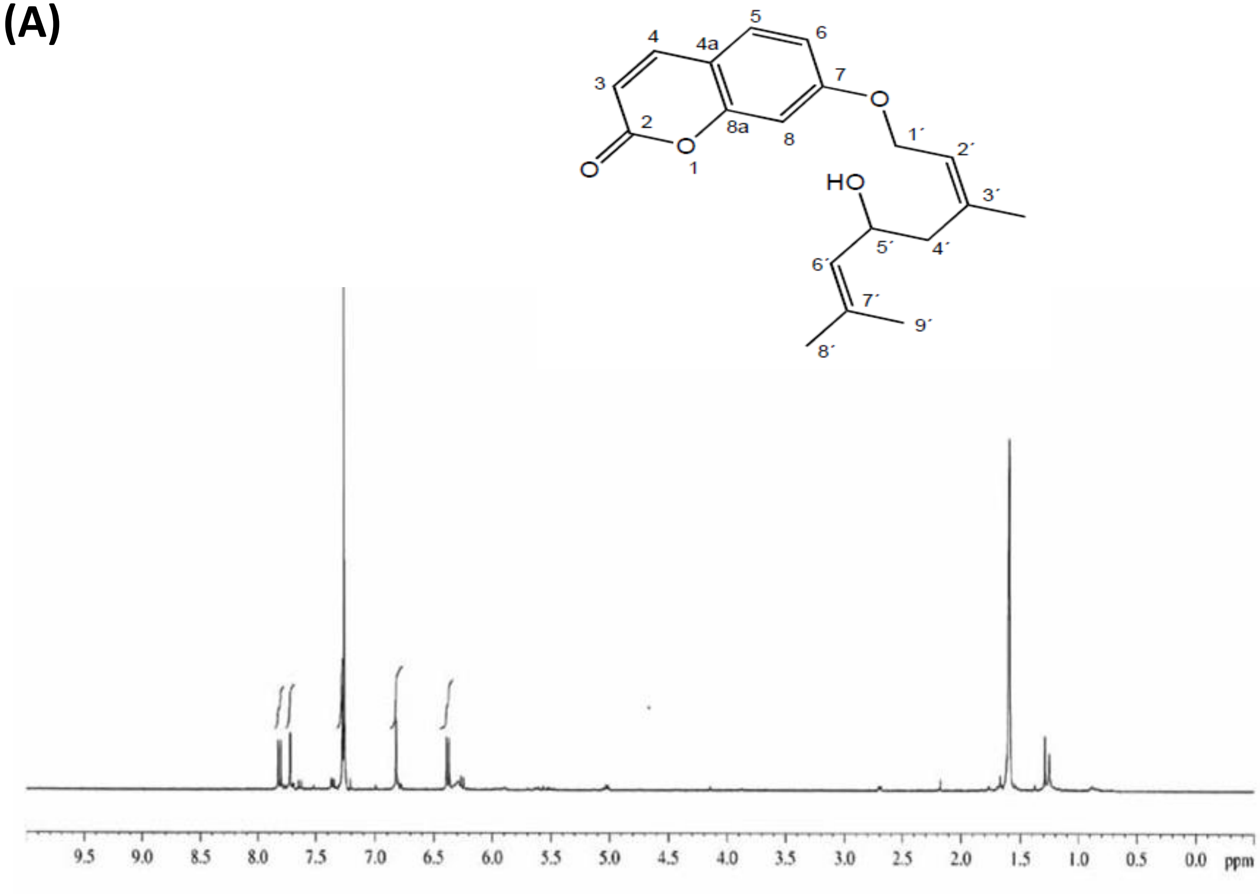

(B)

Position	γH (*J* in Hz)	γ_C
-	-	-
2	-	130.4
3	5.36 (d, 9.6)	124.7
4	4.76 (d, 9.6)	154.3
4a	-	136.5
5	8.36 (s)	123.4
6	-	145.8
7	-	138.6
8	-	121.5
8a	-	153.8
2′	6.68 (d, 2.0)	166.7
3′	7.81 (d, 2.0)	116.8
1″	3.04 (d, 7.2)	75.8
2″	3.68 (dt, 7.2, 1.2)	152.9
3″	-	149.6
4″	1.17 (m)	52.8
5″	5.41 (m)	88.2
6″	2.11 (m)	157.2
7″	-	125.1
8″	3.68 (s)	11.9
9″	3.76 (s)	35.7
10″	3.65 (s)	16.2

Figure S1: NMR spectrum of 5'-hydroxy-aurapten

(A) 1H NMR spectrum of 5'-hydroxy-aurapten (400 MHz, CDCl3).

(B) ^{1}H and ^{13}C NMR spectral data of5'-hydroxy-aurapten in CDCl3
