# Supplementary material for: 5′-hydroxy Auraptene stimulates osteoblast differentiation of bone marrow-derived mesenchymal stem cells via a BMP-dependent mechanism
Source: J Biomed Sci. 2019 Jul 5;26:51. doi: 10.1186/s12929-019-0544-7 (PMC6610929; doi:10.1186/s12929-019-0544-7)
Supplement: Supplementary file 2 — Figure S2. Chemical Structure of 5′-Hydroxy Auraptene. Chemical structure of isolated and purified 7-(5-Hydroxy-3,7-dimethylocta-2,6-dienyloxy)-chromen-2-one. (PDF 201 kb) [file 12929_2019_544_MOESM2_ESM.pdf]

Additional file 2: Figure S2

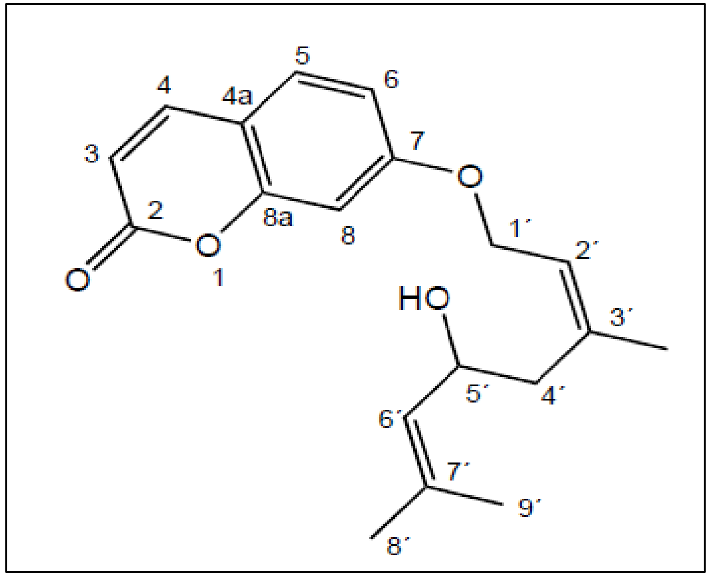

Figure S2: Chemical Structure of 5'-Hydroxy Auraptene
Chemical structure of isolated and purified 7-(5-Hydroxy-3,7-dimethylocta-2,6-dienyloxy)-chromen-2-one
