# Supplementary material for: 5′-hydroxy Auraptene stimulates osteoblast differentiation of bone marrow-derived mesenchymal stem cells via a BMP-dependent mechanism
Source: J Biomed Sci. 2019 Jul 5;26:51. doi: 10.1186/s12929-019-0544-7 (PMC6610929; doi:10.1186/s12929-019-0544-7)
Supplement: Supplementary file 3 — Figure S2. Effect of 5′-HA on gene expression of BMPs antagonists and Smad4. (A) 5′-HA did not affect the mRNA expression of BMPs antagonists including Smurf1/2, Nog, Gremlin1/2 and Chrd, while (B) stimulating the transcription of Smad4 gene as measured by qPCR analysis. (PDF 467 kb) [file 12929_2019_544_MOESM3_ESM.pdf]

Additional file 3: Figure S3

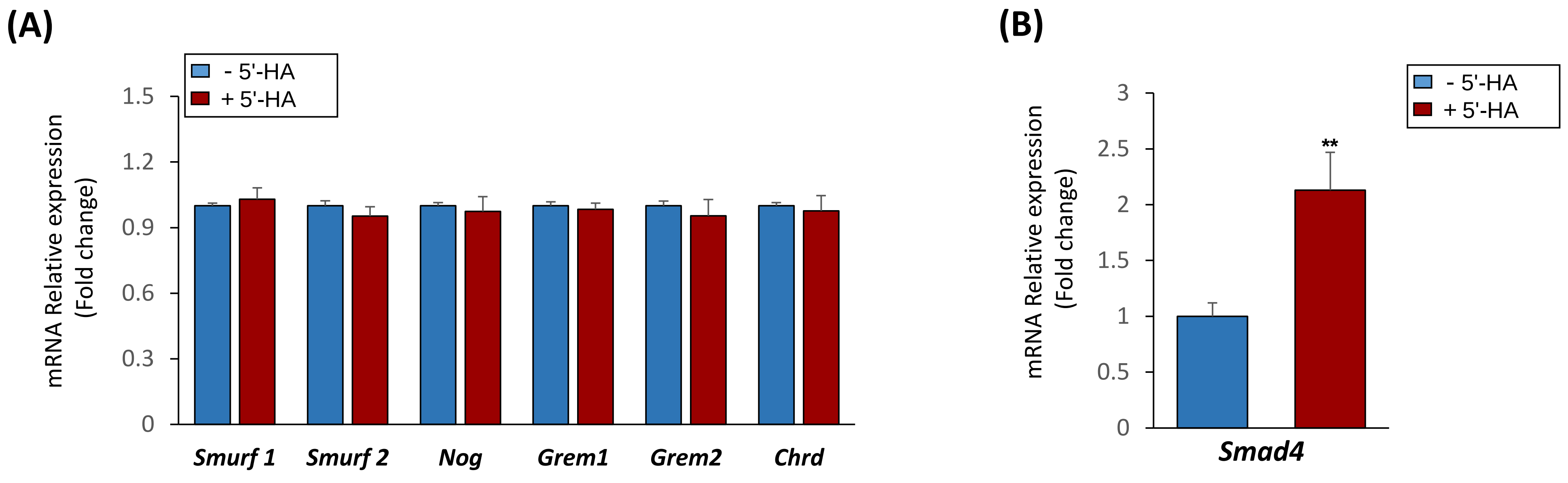

Figure S3: Effect of 5'-HA on gene expression of BMPs antagonists and Smad4

(A) 5'-HA did not affect the mRNA expression of BMPs antagonists including *Smurf1/2, Nog, Gremlin1/2* and *Chrd* , while (B) stimulating the transcription of *Smad4* gene as measured by qPCR analysis. mBMSCs cells were induced to osteoblast differentiation using BMP2 (50 ng/ml) in the absence (- 5'-HA) or the presence of 5'-HA (50 μM) (+ 5'-HA). Each target gene was normalized to reference genes and represented as fold change over non-induced control. Values are mean ± SD of three independent experiments, (*p< 0.05, **p< 0.005 compared to control without 5'-HA).
