# Supplementary material for: 5′-hydroxy Auraptene stimulates osteoblast differentiation of bone marrow-derived mesenchymal stem cells via a BMP-dependent mechanism
Source: J Biomed Sci. 2019 Jul 5;26:51. doi: 10.1186/s12929-019-0544-7 (PMC6610929; doi:10.1186/s12929-019-0544-7)
Supplement: Supplementary file 4 — Table S1. List of primers used for qPCR. List of primer sequences used for qPCR. (PDF 244 kb) [file 12929_2019_544_MOESM4_ESM.pdf]

Supplementary Table 1: List of primers used for qPCR

Gene name	Forward primer 5'-3'	Reverse Primer 5'-3'
β-Actin	GAT ATC GCT GCG CTG GTC GTC	ACG CAG CTC ATT GTA GAA GGT GTG G
Hprt	TCAGTCAACGGGGGACATAAA	GGGGCTGTACTGCTTAACCAG
PPAR-γ	GGG TCA GCT CTT GTG AAT GG	CTG ATG CAC TGC CTA TGA GC
C/ebp-α	AAG CCA AGA AGT CGG TGG A	CAG TCC ACG GCT CAG CTG TTC
aP2	CAA AAT GTG TGA TGC CTT TGT G	CTC TTC CTT TGG CTC ATG CC
Lpl	CTGCTGGCGTAGCAGGAAGT	GCTGGAAAGTGCCTCCATTG
Apm1	GAC GTT ACT ACA ACT GAA GAG C	CAT TCT TTT CCT GAT ACT GGT C
Runx2	AGC AAC AGC AAC AAC AGC AG	GTA ATC TGA CTC TGT CCT TG
Ocn	CAG ACA AGT CCC ACA CAG CA	CTT TAT TTT GGA GCT GCT GT
Alp	GCC CTC TCC AAG ACA TAT A	CCA TGA TCA CGT CGA TAT CC
Opn	GAA ACT CTT CCA AGC AAT TC	GGA CTA GCT TGT CCT TGT GG
Msx2	CCATATACGGCGCATCCTACC	CAACCGGCGTGGCATAGAG
Dlx5	CTGGCCGCTTTACAGAGAAG	CTGGTGACTGTGGCGAGTTA
Smad4	TTTGCTTGGGTCAACTCT	ACTGCACTCCTTTGCCTA
